# AXL receptor tyrosine kinase as a promising anti-cancer approach: functions, molecular mechanisms and clinical applications

**DOI:** 10.1186/s12943-019-1090-3

**Published:** 2019-11-04

**Authors:** Chenjing Zhu, Yuquan Wei, Xiawei Wei

**Affiliations:** 1 Laboratory of Aging Research and Cancer Drug Target, State Key Laboratory of Biotherapy and Cancer Center, National Clinical Research Center for Geriatrics, West China Hospital, Sichuan University, No. 17, Block 3, Southern Renmin Road, Chengdu, Sichuan 610041 People’s Republic of China; 2 Department of Radiation Oncology, Jiangsu Cancer Hospital & Jiangsu Institute of Cancer Research & The Affiliated Cancer Hospital of Nanjing Medical University, 42 Baiziting, Nanjing, 210009 Jiangsu China

**Keywords:** AXL, Receptor tyrosine kinase, Signaling pathway, Inhibitor, Cancer

## Abstract

Molecular targeted therapy for cancer has been a research hotspot for decades. AXL is a member of the TAM family with the high-affinity ligand growth arrest-specific protein 6 (GAS6). The Gas6/AXL signalling pathway is associated with tumour cell growth, metastasis, invasion, epithelial-mesenchymal transition (EMT), angiogenesis, drug resistance, immune regulation and stem cell maintenance. Different therapeutic agents targeting AXL have been developed, typically including small molecule inhibitors, monoclonal antibodies (mAbs), nucleotide aptamers, soluble receptors, and several natural compounds. In this review, we first provide a comprehensive discussion of the structure, function, regulation, and signalling pathways of AXL. Then, we highlight recent strategies for targeting AXL in the treatment of cancer.AXL-targeted drugs, either as single agents or in combination with conventional chemotherapy or other small molecule inhibitors, are likely to improve the survival of many patients. However, future investigations into AXL molecular signalling networks and robust predictive biomarkers are warranted to select patients who could receive clinical benefit and to avoid potential toxicities.

## Introduction

Cancer is not a single-cell disease but rather the result of complex interactions of tumour cells with surrounding matrix and immune cells. In recent years, molecular targeted therapy for cancer has been a research hotspot. Tyrosine kinase inhibitors (TKIs) targeting epidermal growth factor receptor (EGFR), vascular endothelial growth factor (VEGF) and platelet-derived growth factor receptor (PDGFR) have been evaluated in clinical trials with promising results, which prompted the search for additional diagnostic and prognostic biomarkers [1–3]. AXL has emerged as a novel biomarker due to its role in biological processes and tumourigenesis [4]. AXL is a member of the TAM family that includes TYRO3, AXL and MER. Growth arrest-specific protein 6 (GAS6) serves as a ligand for AXL with high binding affinity. GAS6/AXL signalling functions as an important pathway driving cancer cell survival, proliferation, migration and invasion, which makes AXL a potential target in cancer treatment [5, 6].

## The structure and function of AXL

**Fig. 1:**
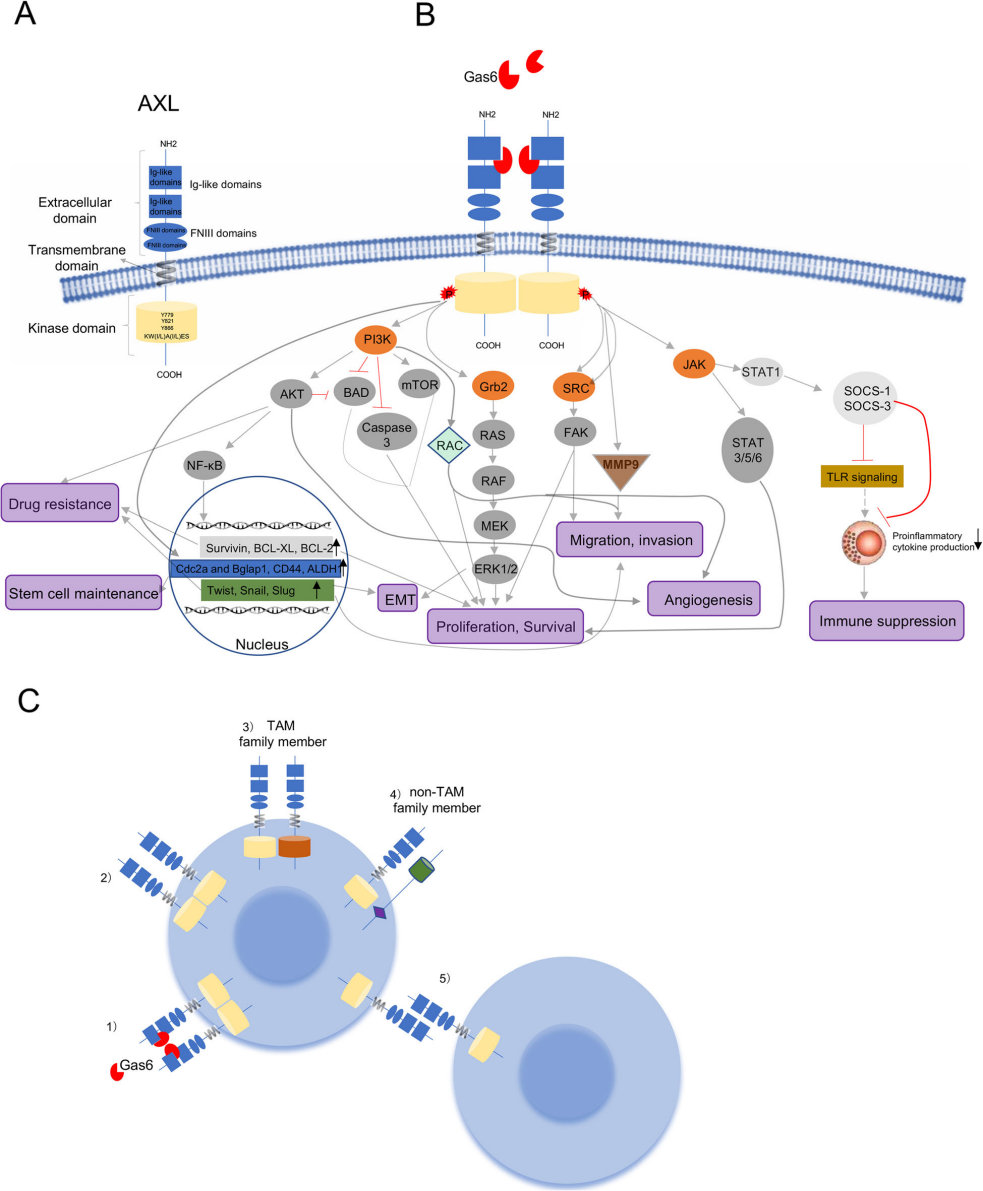
Basic structure, signaling pathways and activation of AXL. (**a**) Schematic diagram representing the structure of AXL receptor tyrosine kinase. AXL is composed of two immunoglobulin (Ig)-like repeats and two fibronectin type III (Fro III)-like repeats, a transmembrane domain and an intracellular kinase domain. (**b**) AXL signaling networks upon classical GAS6-mediated activation function in proliferation and survival, migration and invasion, epithelial-to-mesenchymal transition (EMT), angiogenesis, resistance to therapy, immune suppression, and stem cell maintenance. (**c**) AXL activation patterns: 1) classical GAS6 ligand-dependent dimerization; 2) Gas6 ligand-independent dimerization; 3) heterophilic dimerazation of AXL with a TAM family member like MER or TYRO3; 4) heterophilic dimerazation of AXL with a non-TAM family protein; and 5) ligand-independent activation of AXL through transcellular homophilic binding

The gene *AXL*, located at chromosome 19q13.2, was first identified in patients with chronic myeloid leukaemia (CML) [7]. The word *AXL*, coming from the Greek word “anexelekto”, means uncontrolled. The protein encoded by the gene *AXL*, called AXL (UFO, ARK, Tyro7, or JTK11), is a member of the TAM family of receptor tyrosine kinases (RTKs). AXL is composed of an extracellular, transmembrane and intracellular domain [8]. The extracellular structure consists of two immunoglobulin (Ig)-like repeats and two fibronectin type III (Fro III)-like repeats that resemble neural cell adhesion molecules (NCAMs) [9]. The Ig motifs are involved in the binding of AXL with its ligand Gas6 under regulation by Fro III [10]. The intracellular domain is critical for auto-phosphorylation and subsequent kinase activity [11]. The TAM family-specific KW(I/L)A(I/L)ES sequence resides within this intracellular domain, which shares homology with AXL-related RTKs, such as RET [12], and plays an important role in tyrosine kinase activity (Fig. 1a). The TAM family is widely expressed in normal cells and tissues, such as monocytes, platelets, endothelial cells, hippocampus, cerebellum, heart, and liver [13–20], wherein it regulates cell survival, the non-inflammatory clearance of apoptotic cells by phagocytic cells, natural killer cell differentiation, platelet aggregation, etc. [10, 21–23].

## The regulation of *AXL* gene expression

The synthesis of *AXL* is regulated at many levels. Five transcription factors act on the *AXL* promotor, including activator protein 1 (AP1) [24], Sp1/Sp3, YAP/TAZ/TEAD, hypoxia inducible factor 1α (HIF1α) [25] and myeloid zinc finger 1 protein (MZF-1) [26]. Activation of TLR signalling upregulates AXL mRNA in dendritic cells and macrophages [27, 28]. The transcription process is also feedback controlled by other RTKs. In non-small-cell lung cancer (NSCLC) and head and neck squamous cell carcinoma (HNSCC), activated EGFR pathways and downstream MEK/ERK signalling promote *AXL* mRNA expression through the JUN transcription factor [29]. In urothelial carcinoma, *AXL* mRNA expression is induced after activation of MET and downstream MEK/ERK signalling [30]. *AXL* mRNA expression is inhibited by two microRNAs (miRNAs), miR-34a and miR-199a/b [31, 32]. In addition, *AXL* mRNA expression is subject to epigenetic modification, including histone acetylation and histone/DNA methylation [33, 34]. Although full-length AXL contains 894 amino acids and encodes a predicted protein of 98 kDa, the actual observed molecular weight ranges from 100 to 140 kDa due to post-transcriptional regulation of the activated AXL receptor by glycosylation, phosphorylation and multiple sites of monoubiquitination [35–39].

In adults, AXL expression is relatively low [40, 41], but aberrant expression of Gas6/AXL has been shown in a number of human malignancies, including breast cancer, chronic lymphocytic leukaemia (CLL), NSCLC, pancreatic cancer, glioblastoma, melanoma, renal cell carcinoma (RCC), prostate cancer, and oesophageal cancer [4, 23, 42–55], and this altered expression is associated with disease progression and shortened overall survival (OS). In various in vivo breast cancer models, AXL expression was found to be higher in metastatic nodules than in primary tumours, and downregulating AXL with miRNAs inhibited downstream AKT phosphorylation and diminished the motility, metastasis and invasion of tumour cells [47]. Given the role of AXL in cancer development, progression and drug resistance, AXL holds great promise as a prognostic biomarker and therapeutic target.

## Gas6/AXL axis and its role in tumour development and progression

Common ligands for the TAM family include Gas6, protein S, Tubby, Tubby-like protein 1 (TULP-1) and Galectin-3 [56, 57]. Studies on Gas6 and protein S are abundant, as these two ligands were the first to be discovered. Gas6 can bind to all three members of the TAM family, whereas protein S binds only to MER and TYRO3. The affinity of Gas6 for AXL is 3–10 times higher than that for the other two members in the family [8, 58]. Gas6 is encoded by growth arrest-specific gene 6 (*Gas6*) and belongs to the vitamin K-dependent protein family [59]. Upon high-affinity binding to its ligand Gas6, the AXL receptor undergoes homo-dimerization and subsequent trans-autophosphorylation within the intracellular kinase domain, thus recruiting adaptor molecules and effector proteins containing Src homology 2 (SH2) or other phosphotyrosine-binding domains (PTBs) and activating downstream signalling pathways [60, 61]. Six phosphorylation sites have been found in AXL, Tyr698, Tyr702, Tyr703, Tyr779, Tyr821 and Tyr866. Three N-terminal tyrosine residues, 779, 821 and 866, are related to auto-phosphorylation and AXL activation, while the other three C-terminal sites are rather conserved among the TAM receptors and indispensable for complete functions of the kinase [10]. A study on a EGFR-AXL chimeric receptor has revealed that the phosphatidylinositol 3-kinase (PI3K)/AKT/mTOR signalling pathway is induced by binding of Tyr779 and Tyr821 to p85, and the MEK/ERK cascade is activated by binding of phosphorylated Tyr821 to GRB2 [62]. In addition, phosphorylated Tyr821 and Tyr866 can bind to SRC, LYK and PLCα and activate protein kinase C and STAT [4, 10, 15, 61]. Therefore, the PI3K/AKT/mTOR, JAK/STAT, NF-κB, and RAS/RAF/MEK/ERK signalling pathways function as important downstream pathways for the Gas6/AXL axis and play major roles in tumour cell survival, anti-apoptosis signalling, mitogenesis, migration, invasion, drug resistance, angiogenesis and the tumour-host relationship [63–66] (Fig. 1b).

In addition to Gas6-dependent AXL activation, AXL might also be activated via Gas6-independent mechanisms [62]. Overexpression of AXL leads to aggregation of AXL extracellular domains or ligand-independent homo-dimerization, thereby leading to receptor activation [13, 53]. Moreover, the broad homology AXL shared with other TAM family members, as well as with other non-TAM RTKs such as fibroblast growth factor receptor (FGFR), EGFR, PDGFR and MET [4], encourages the formation of heterodimers and the activation of AXL-dependent signalling [67, 68] (Fig. 1c). In multiple tumour tissues, aberrant AXL expression is related to the extent of malignancy, metastasis and a poor prognosis.

### AXL promotes cell proliferation

AXL potentially drives cell proliferation through effector molecules in the PI3K/AKT/mTOR, RAS/RAF/MEK/ERK, JAK/STAT, and NF-κB signalling pathways [10, 69]. AXL fosters cell survival by regulating NF-κB nuclear translocation, increasing the expression of anti-apoptotic markers (survivin, BCL-2, and BCL-XL) and reducing the activity of pro-apoptotic proteins (BAD and caspase-3) [45, 52, 70]. Knockdown of *AXL* with short hairpin RNA (shRNA) reduces Ki67 expression and increases apoptosis-related protein levels [18, 71]. In CLL, the suppression of AXL promotes apoptosis with reduced levels of the anti-apoptotic protein MCL-1 [72]. Blockade of Gas6/AXL signalling pathway is sufficient to suppress ectopic and orthotopic glioma growth, leading to a marked prolongation of survival [73]. Similar results have been reported in prostate cancer, mesothelioma, lung adenocarcinoma and colorectal cancer [74, 75]. Thus, AXL may be implicated in protecting tumour cells from the apoptotic effects of numerous drugs.

### AXL mediates migration and invasion

AXL was shown to be a driving force in the spread of tumours in both in vivo and in vitro studies. AXL activity is critical for cell migration phenotypes, including the increase in the GTP-binding proteins Rho and Rac [76] and the formation of filopodia [77]. The overexpression of AXL in cells with low metastatic colonization potential leads to augmented migratory and invasive abilities [78, 79]. It has been shown that AXL mediates Yes-associated protein (YAP)-dependent oncogenic functions that potentiate migration and invasion in hepatocellular carcinoma (HCC) [80]. In oesophageal adenocarcinoma (EAC) cell lines, AXL is responsible for the peripheral distribution of lysosomes and the secretion of cathepsin B, which promote cell invasion [55]. Matrix metalloproteinase 9 (MMP9) has been identified as a required factor for AXL-mediated invasion in vitro and in vivo [76, 78]. AXL activation promotes the expression of p-AKT and MMP9 through activation of NF-κB and Brg-1 [78, 81, 82]. Depletion of TIG1, which stabilizes AXL and prevents AXL from degradation, leads to a reduction in MMP9 expression in inflammatory breast cancer cell lines, reducing the in vitro migration and invasion of cells [71, 83]. Attenuation of the AXL signalling axis with the anti-AXL monoclonal antibody (mAb) 20G7-D9 dramatically reduces the number of bone metastases after intracardiac injection of breast cancer cells [84]. In HCC, downregulation of AXL by shRNA inhibits cell invasion through the PI3K/AKT-P21-activated kinase-1 (PAK1) signalling pathway. The reduced migration and invasion of cancer cells upon RNA interference (RNAi)-mediated knockdown of *AXL* or blockade of AXL signalling were also reported in liposarcoma, pancreatic cancer, lung adenocarcinoma, breast cancer and thyroid cancer [69, 76, 85–87], thus demonstrating the role of AXL signalling as a good target for conferred migratory and invasive properties.

### AXL affects epithelial-mesenchymal transition (EMT)

Evidence supporting a pro-tumourigenic role for AXL in promoting EMT has recently been described in multiple studies. EMT is a reversible event in which cells undergo a transition from an epithelial phenotype to a mesenchymal phenotype through a special programme, and this transition is critical for foetal development and wound healing [88–90]. Cell-cell adhesion in normal epithelial cells helps maintain tissue integrity; on the other hand, mesenchymal cells are migratory and invasive [91]. Characteristic protein changes during EMT include a reduction in epithelial markers such as E-cadherin and an increase in mesenchymal markers such as N-cadherin, Snail, Vimentin, Slug, α-catenin and α-SMA [92, 93]. AXL activation drives EMT and enables cells to retain a mesenchymal phenotype [94]. In human breast cancer epithelial cells, transfection of *SLUG* and *SNAIL* into MCF10A cells is associated with increased expression of AXL, which induces the loss of epithelial-type morphology and the gain of mesenchymal-related markers [47]. Cells may respond to *AXL* deprivation by losing the expression of EMT-associated transcription factors (i.e., Slug, Zeb1, snail and Twist) [76, 95, 96], stimulating E-cadherin expression and cell-cell adhesion, and attenuating the activity of TGFâ-R and WNT signalling, thereby reversing to an epithelial-type morphology [44, 97, 98].

### AXL in angiogenesis

Angiogenesis is a normal physiological process during foetal development, growth, wound healing, tissue reconstruction and repair [99]. However, it may also provide oxygen, nutrients and essential hormones to tumour cells, contributing to tumour growth, expansion and metastasis [100]. In addition to their oncogenic functions, TAM family members play important roles in vessel integrity and promoting angiogenesis with VEGF, fibroblast growth factor (FGF) and platelet-derived growth factor (PDGF) [10, 28, 101–103]. AXL is broadly expressed in endothelial cells and vascular smooth muscle cells; it promotes the stabilization of aggregated platelets, survival of endothelial cells and remodelling of endothelial barriers in wound healing and vessel impairment [104]. The autocrine and paracrine loops of AXL and its ligand Gas6 may enhance the activity and proliferation of endothelial cells, regulate the function of integrin, and promote the migration and survival of endothelial cells and tumour cells through RAC and AKT signalling [8]. AXL catalytic activity induces human umbilical vein endothelial cell (HUVEC) growth, migration and tube formation [105]. Knockdown of either the *AXL* or *Gas6* gene will impair endothelial tube formation and functional circulation [106, 107]. However, Gallicchio et al. [108] implied an anti-angiogenic role for AXL by showing that the Gas6/AXL axis might antagonize VEGFR2-dependent angiogenesis. Studies have demonstrated that AXL expression is associated with antiangiogenic resistance, while the combination of AXL inhibitors with antiangiogenic agents could reduce vessel density in renal cell carcinoma patient-derived xenografts [109–111]. The complex role of AXL in angiogenesis under normal or pathological conditions needs to be further discussed.

### AXL is associated with drug resistance in cancer treatment

Drug resistance is a thorny problem and a major hindrance during cancer management that often leads to treatment failure or disease recurrence. Numerous examples of the association of AXL expression with treatment resistance have been reported in prostate, breast, ovarian, colorectal and lung cancers [29, 48, 51, 65, 112, 113]. AXL could cause innate or acquired resistance to chemotherapy, immune therapy, molecular targeted therapy or even radiation therapy [98, 114–118]. Molecular targeted therapy leads to an increase in AXL expression, which confers refractoriness or an insufficient response of cells to ERK, BRAF, PI3Kα, ALK, EGFR and VEGFR inhibitors [29, 114, 117, 119, 120]. The suppression of AXL, whether by genetic knockdown or pharmacological inhibition, is effective in circumventing chemoresistance to certain drugs in an otherwise resistant cell line. For example, AXL was identified as a “tyrosine kinase switch”: overexpression of AXL/Gas6 and low KIT expression were found in an imatinib (KIT/PDGF inhibitor)-resistant gastrointestinal stromal tumour (GIST) model [118]. In cisplatin-resistant ovarian cancer cells, *AXL* mRNA expression was twice as high as that in cisplatin-sensitive cells [121]. Selective silencing or inhibition of AXL resensitized CML cells to imatinib [49] and prostate cancer cells to docetaxel with reduced ATP-binding cassette B1 (ABCB1) levels [98]. In radiation-resistant HNSCC cell lines, marked AXL overexpression was found in cancer cell xenografts and patient-derived xenografts (PDXs), whereas resensitization to chemotherapy and radiation was achieved after *AXL* knockdown [114, 122].

Over the years, many studies have investigated the mechanisms through which AXL induces drug resistance. These underlying mechanisms might involve crosstalk between AXL and other RTK family members. As mentioned previously, AXL can heterodimerize with non-TAM RTKs such as EGFR, MET and PDGF, which helps avoid the effects of certain RTK inhibitors. For instance, the hetero-interaction between AXL and human epidermal growth factor receptor 2 (HER2) leads to downstream PI3K/AKT and ERK signalling and allows cells to evade the inhibitory effects of lapatinib in HER2-positive breast cancer [123]. Moreover, in PI3K inhibitor-resistant squamous cell carcinoma, AXL binds to EGFR and activates the PLCα/PKC/mTOR signalling pathway to maintain tumour progression [48]. AXL expression also sustains the effects of PI3K/AKT and MEK/ERK, and positive feedback from MEK/ERK induces *AXL* transcription through JUN. In acute myeloid leukaemia (AML), AXL is associated with upregulated BCL-2 and Twist and participates in the malignant progression of cells, inducing EMT and drug resistance [45]. These findings indicate that AXL is a promising target for the salvage treatment of cancer recurrence.

### AXL regulates the immune response

TAM family members are important negative inflammatory mediators inhibiting certain signalling pathways that activate dendritic cells, natural killer cells and macrophages [27, 103, 124, 125], attenuating their ability to eliminate metastases [27, 126, 127]. It was reported that TAM receptors could suppress cytokine production and the TLR-dependent inflammatory response through hijacking pro-inflammatory signals, thereby serving as a feedback mechanism to prevent autoimmune responses [27].

Given the recent growing interest in immune checkpoint blockade, the role of AXL in immune surveillance has garnered much attention. AXL activation is involved in immune evasion through the upregulation of BCL-2 and Twist, the suppression of TLR inflammatory signalling and natural killer cells, and the limited expression of pro-inflammatory cytokines [4, 45], and AXL loss-of-function enhances chronic inflammation and autoimmunity [23, 27, 128–131]. The combined deletion of *AXL* and *MER* has been shown to increase the risk of colitis and colitis-associated cancer [132]. The role of AXL in radioresistant and checkpoint immune-resistant tumours has been described, and the mechanism is thought to involve the ability of AXL to suppress antigen presentation through MHC-I and to enhance myeloid-supporting cytokines and chemokines, resulting in a limited initial immune response [133]. These findings are further supported by the recent genomic and transcriptomic data from metastatic melanoma patients suggesting that AXL overexpression might influence innate sensitivity or cause resistance to anti-PD-1 therapy [115]. Interestingly, the immunomodulatory function of AXL in oncogenesis is paradoxical. Diminishing AXL and MER signalling in normal mouse tissue induces the production of inflammatory cytokines, which favour a pro-malignancy environment [131]. Thus, the mechanisms by which AXL inhibitors modulate the immune response will be important to decipher for additional insight into anticancer therapeutic approaches.

### AXL is related to stem cell maintenance

Cancer stem cells (CSCs) are a small subpopulation of cells that reside within the tumour; possess the capabilities of self-renewal, differentiation and tumourigenicity; and exert an immense influence on tumour resistance, recurrence and metastasis [134, 135]. AXL expression is found in human gliomas with high expression of EZH2, which plays a crucial role in stem cell maintenance [33]. Additionally, AXL is correlated with the expression of stem cell marker genes such as Isl1, Cdc2a, Bglap1, CD44 and ALDH1, which increase the tumourigenicity of breast cancer stem cells [96] and enhance the resistance of cutaneous squamous cell carcinoma to chemotherapy [95]. Targeting AXL holds great therapeutic potential to diminish WNT/â-catenin and TGFâR signalling and sphere formation ability, and therefore, to repress cancer resistance and progression [136–138].

## AXL-targeted therapies

**Fig. 2:**
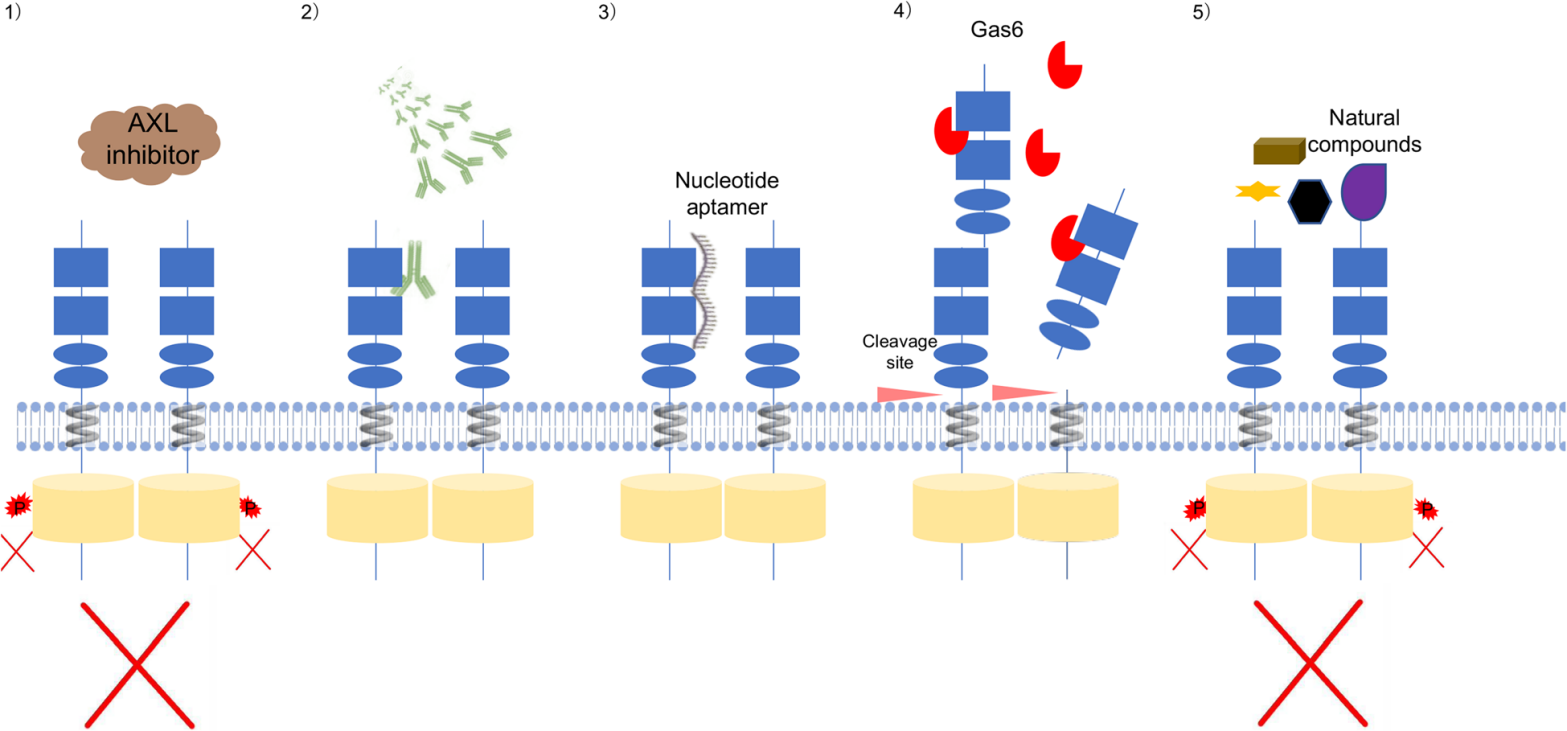
Different therapeutic agents targeting AXL have been developed, including 1) small molecule inhibitors that block AXL auto-phosphorylation and kinase activities; 2) anti-AXL monoclonal antibodies (mAbs); 3) nucleotide aptamers; 4) soluble receptors; and 5) several natural compounds

Because of the pleiotropic role of AXL in tumour pathophysiology and drug resistance, AXL represents a promising therapeutic target in the management of cancer. AXL inhibitors have been shown to potentiate tumour cell apoptosis and suppress migration and invasion [43]. Targeting AXL enhances the therapeutic efficacy of chemotherapy and other small-molecule inhibitors, such as VEGF, EGFR, PI3K, PARP, and HER2 inhibitors [54, 123, 139]. Different therapeutic agents have been developed over the decades, including small molecule inhibitors, anti-AXL mAbs, nucleotide aptamers, soluble receptors, and several natural compounds (Fig. 2).

### Small molecule inhibitors

Most small molecules that have inhibitory effects on AXL were not synthesized to act primarily on AXL; thus, the AXL inhibitory activity is not as potent as the inhibitory activity against other kinases [46, 140, 141]. However, BMS-777607, originally designed as a MET-branded inhibitor, was approximately three times more potent against AXL than MET [142]. AXL-specific inhibitors were also developed with high selectivity, including SGI-7079, TP-0903, BGB324, DP3975 and NA80xl. All compounds described here are ATP-competitive inhibitors, either the most commonly observed type I inhibitors that preferentially occupy the ATP-bound pocket of the kinase in the active “aspartate-phenylalanine-glycine (DFG)-in” conformation, or type II inhibitors that prefer the inactive DFG-out conformation [143, 144] and that are reported to be not intrinsically more selective than type I inhibitors [145]. Several AXL inhibitors have been considered promising upon emerging from preclinical studies and are thus advanced into different stages of clinical investigation [146–153].

#### Type I AXL inhibitors (Table 1)

##### BGB324 (Bemcentinib, R428; Rigel Pharmaceuticals/BerGenBio)

R428, a specific and highly selective AXL inhibitor, acts on AXL at nanomolar concentrations (IC50 = 14 nM) with 100-fold higher affinity for AXL over ABL in cellular assays [46, 154]. R428 blocks the catalytic activities of AXL and reduces AXL and p-AXL expression [46, 58, 155]. When used alone or in combination with cytotoxic agents to treat AML cells, R428 inhibits the AKT and MAPK pathways through the upregulation of Puma and subsequently suppresses BCL-2 [156]. R428 has been shown to recover drug sensitivity in many models of acquired resistance; for example, R428 inhibits erlotinib-resistant head and neck cancer cell growth and migration [114, 116]. In 2014, R428 was the first AXL-specific TKI to enter clinical trials, and this drug is now in phase I/II clinical trials for AML, myelodysplastic syndromes (MDS), triple-negative breast cancer (TNBC), metastatic melanoma, pancreatic cancer, and NSCLC either alone or in combination with other chemotherapy regimens (NCT02424617, NCT02922777, NCT03184558, NCT02488408, NCT03184571, NCT03649321, NCT03824080, and NCT02872259).

**Table 1:** Summary of the basic profile of type I AXL inhibitors and the related ongoing clinical trials

Drug	Developer	Target(s)^a^	IC50 for AXL	Clinical Trial No.^b^	Phase of approval	Indications	Monotherapy/Combinations	Adverse events	Status
BGB324 (R428)	Rigel Pharmaceuticals/BerGen BIO	AXL (selective)	IC50 (in vitro) = 14 nM IC50 (in cells) = 14 nM	NCT02424617	I/II	NSCLC	+ Erlotinib	Not reported	Recruiting
				NCT03184558	II	TNBC	+ Pembrolizumab		Recruiting
				NCT02488408	Ib/II	AML, MDS	± Cytarabine/decitabine		Recruiting
				NCT02872259	Ib/II	Metastatic melanoma	+ Pembrolizumab; + Dabrafenib and trametinib		Recruiting
				NCT03649321	Ib/II	Pancreatic cancer	± Nab-paclitaxel/gemcitabine/cisplatin		Recruiting
				NCT03824080	II	MDS	Monotherapy		Recruiting
TP-0903	Tolero Pharmaceuticals	AXL (selective)	IC_50_ (in vitro) = 27 nM IC50 (in cells) = 222 nM	NCT03572634	I/II	CLL	± Ibrutinib	Not reported	Not yet recruiting
				NCT02729298	I	Advanced solid tumors	Monotherapy		Recruiting
Crizotinib (PF-02341066, marketed as [Xalkori])	Pfizer	MET, ALK, RON, AXL	IC_50_ (in vitro) = 294 nM IC_50_ (in vivo) = 322 nM	NCT02737501	III	ALK-positive locally advanced or metastatic NSCLC	Crizotinib vs. Brigatinib	Abdominal pain, headache, pyrexia, pain in extremity, nausea	Active, not recruiting
				NCT02223819	II	Uveal melanoma	Monotherapy		Active, not recruiting
				NCT02435108	II	MET-positive gastric cancer	Monotherapy		Completed
				NCT02207504	I	Castration-resistant prostate cancer	+ Enzalutamide		Active, not recruiting
Bosutinib (SKI-606, Bosulif®)	Pfizer	ABL, SRC, AXL	IC_50_ (in vitro) = 174 nM	NCT02228382	IV	Previously treated Ph + CML	Monotherapy	Diarrhea, rash, liver enzyme elevations	Active, not recruiting
				NCT03106779	III	CML	Bosutinib vs. ABL001		Recruiting
				NCT01331291	II	Glioblastoma	Monotherapy		Completed
				NCT00319254	II	Breast cancer	Monotherapy		Completed
				NCT03023319	I	Metastatic solid tumors	+ Pemetrexed		Recruiting
Gilteritinib (ASP2215)	Astellas Pharma/Kotobuki Pharmaceutical	FLT3, AXL	IC_50_ (in vitro) = 0.73 nM	NCT02927262	III	Relapsed or treatment refractory FLT3 mutated AML	Gilteritinib or placebo	Febrile neutropenia, anemia, thrombocytopenia, sepsis, pneumonia, diarrhea, fatigue, elevated aspartate aminotransferase and alanine aminotransferase	Recruiting
				NCT02456883	I	Advanced solid tumors	Monotherapy		Completed
				NCT02495233	I/II	NSCLC	+ Erlotinib		Terminated
S49076	Servier	MET, MER, AXL FGFR3	IC_50_ (in vitro) = 7 nM IC_50_ (in cells) = 56 nM	ISRCTN00759419	I	Advanced solid tumors	Monotherapy	Peripheral oedema and hypoalbuminaemia	Completed
Amuvatinib (MP-470)	Astex Pharmaceuticals	KIT, AXL, PDGFR1, FLT3, RET	IC_50_ (in vitro) = 10 nM	NCT01357395	II	SCLC	Monotherapy	Fatigue, alopecia, diarrhea, nausea, anorexia, neutropenia, anemia, thrombocytopenia, leukopenia	Completed
				NCT00894894	I	Solid tumors	Monotherapy		Completed
				NCT00504205	Not Applicable	Unresectable or metastatic solid tumor or lymphoma	Monotherapy		Terminated
Sunitinib (SU11248, marketed as Sutent)	Pfizer	PDGFR, VEGFR2, FLT3, KIT, AXL	IC50 (in vitro) = 5 nM	NCT00706706	IV	Metastatic RCC	Monotherapy	Diarrhea, fatigue, hypertension, palmar-plantar erythrodysesthesia, and hematologic adverse events	Completed
				NCT02691793	IV	Refractory Solid Tumors	Monotherapy		Recruiting
				NCT01525550	IV	Pancreatic neuroendocrine tumor	Monotherapy		Completed
				NCT00793871	IV	GIST	Monotherapy		Completed
				NCT00794950	II	Urothelial carcinoma	Monotherapy		Active, not recruiting
				NCT01718327	II	Unresectable and advanced cholangiocarcinoma	Monotherapy		Completed
				NCT01824615	II	Ovarian cancer	Monotherapy		Completed
				NCT02623127	II	Thymic carcinoma	Monotherapy		Completed
				NCT00372775	II	NSCLC	Monotherapy		Completed
				NCT01498835	I	Soft tissue sarcoma	Monotherapy		Completed
SNS314 2-D08	Sunesis Pharmaceuticals	Aurora A/B/C, Trk A/B, FLT4, Fms, Axl	IC50 (in vitro) = 84 nM	NCT00519662	I	advanced solid tumors	Monotherapy	Not reported	Completed
		AXL, IRAK4, ROS1	IC50 (in vitro) = 0.49 nM	–	–	–	–	–	Preclinical
UNC2025	University of North Carolina	MER, FLT3, AXL, Tyro3	IC50 (in vitro) = 14 nM	–	–	–	–	–	Preclinical
SGI-7079	Tolero Pharmaceuticals/Astex Pharmaceuticals	AXL (selective)	IC50 (in vitro) = 58 nM IC50 (in vivo) < 1 uM	–	–	–	–	–	Preclinical
UNC569		MER, AXL, Tyro3	IC50 (in vitro) = 37 nM	–	–	–	–	–	Preclinical
NA80x1		AXL (selective)	IC50 (in vitro) = 12.67 ± 0.45 μM, IC50 (in vivo) = 4.11 ± 1.47uM	–	–	–	–	–	Preclinical
DP-3975	Deciphera Pharmaceuticals, LLC	AXL (selective)	IC50 (in vitro) = 100 nM ∼ 2 uM	–	–	–	–	–	Preclinical

*Abbreviations*: *NSCLC* non-small cell lung cancer, *TNBC* triple-negative breast cancer, *AML* acute myeloid leukemia, *MDS* myelodysplastic syndrome, *CLL* chronic lymphocytic leukemia, *CML* chronic myelogenous leukemia, *FLT3* FMS-like tyrosine kinase 3, *GIST* gastrointestinal stromal tumors, *RCC* renal cell carcinoma, *SCLC* small cell lung cancer

^a^In the order of inhibition potency

^b^All the relevant information of clinical trials can be found on the public clinical trial registry website (clinicaltrials.gov). Here is a partial list of all the relevant clinical trials

##### TP-0903 (Tolero Pharmaceuticals)

TP-0903 is a novel selective AXL inhibitor with an in vitro IC50 of 27 nM [157, 158]. TP-0903 disrupts the phosphorylation of AXL; reverses EMT; enhances the depletion of anti-apoptotic proteins such as MCL-1, XIAP, and BCL-2; and induces dose-dependent CLL cell death [72, 159]. Moreover, it also enhances neuroblastoma cell sensitivity to conventional chemotherapy [160]. TP-0903 is currently being evaluated for safety, pharmacokinetics, pharmacodynamics, and antitumour activity in patients with CLL and refractory solid tumours (NCT03572634 and NCT02729298).

##### Crizotinib (PF-02341066, marketed as Xalkori; Pfizer)

As a multi-target TKI, crizotinib inhibits ALK, MET, RON and AXL [161]. Early preclinical testing of crizotinib revealed potent in vitro and in vivo antitumour effects in a variety of malignancies, including gastric carcinoma, NSCLC, RCC, prostate carcinoma, malignant glioblastoma, anaplastic large cell lymphoma (ALCL), and osteosarcoma [162–165], which prompted abundant clinical research. In phase I and II studies, crizotinib resulted in significant and rapid improvements in treatment responses in patients with *ALK*-positive NSCLC and was generally well tolerated [166]; therefore, crizotinib was approved by the FDA to treat patients with ALK-positive metastatic NSCLC. Later, it was further approved for the treatment of metastatic NSCLC patients with ROS1 gene alterations [167]. However, the unavoidable acquired resistance to first-line crizotinib is a major problem to be managed in patients with ALK-positive NSCLC [168]. Recently, clinical trials have been initiated to compare crizotinib and other ALK inhibitors (NCT02737501) and to assess crizotinib in combination with other agents, such as immune checkpoint inhibitors, mTOR inhibitors, and antiangiogenesis drugs (NCT02292550, NCT02321501, NCT02521051, NCT01998126, NCT02511184, and NCT02393625).

##### Bosutinib (SKI-606, Bosulif®; Pfizer)

Originally developed as an inhibitor of SRC and ABL kinases, the second-generation TKI bosutinib also has potent inhibitory activity against AXL auto-phosphorylation [79], accompanied by the suppression of Slug expression, stabilization of cell-to-cell adhesion and increased membrane localization of â-catenin [141, 169]. Treatment with bosutinib induces the dose- and time-dependent induction of apoptosis in CLL B cells [154] and suppresses the motility and invasiveness of HCC and breast cancer cells [79, 170]. The administration of bosutinib causes the regression of K562 and colorectal xenografts in nude mice [171, 172]. Bosutinib was reported to have significant clinical activity in CML patients who were resistant/intolerant to prior TKIs and was generally well tolerated in these populations in phase I/II clinical trials [173]; thus, it was approved for use in CML at 400 mg q.d. as first-line therapy and at 500 mg q.d. in patients who have failed prior imatinib, nilotinib, or dasatinib [174, 175]. Bosutinib is currently in multiple phase I-IV clinical trials for the treatment of several cancer types, including breast cancer, CML, NSCLC, and glioblastoma.

##### Gilteritinib (ASP2215; Astellas Pharma/Kotobuki Pharmaceutical)

Gilteritinib is a novel, highly specific, dual FMS-like tyrosine kinase 3 (FLT3)/AXL inhibitor that has demonstrated robust antileukaemic activity in patients with relapsed/refractory AML. AXL is known to promote constitutively active FLT3 and to be responsible for resistance to FLT3 inhibitors [176, 177]. Targeting AXL and FLT3 with gilteritinib translated to tumour regression and reduced proliferation in FLT3 mutation-positive cellular and mouse models of AML [178]. Promising early phase I/II trial data of gilteritinib demonstrated antileukaemic activity and acceptable side effects in relapsed/refractory AML patients from the USA, Germany, Italy and Japan (NCT02181660 and NCT02014558) [179, 180]; thus, it was approved in Japan and the USA for the treatment of relapsed or refractory AML with FLT3 mutation [181]. The clinical development of gilteritinib for advanced solid tumours is also underway in several countries worldwide (NCT02456883, NCT02561455, and NCT02622932).

##### S49076 (Servier)

S49076 is described as a novel ATP-competitive TKI of MET, AXL, and FGFR1/2/3 [182]. S49076 exerts its cytotoxic activity at low doses on MET- and FGFR2-dependent cells, while it blocks the proliferation of MET-independent cells at higher but clinically relevant doses through targeting Aurora B [183]. In tumour xenograft models, S49076 enhances the antitumour efficacy by synergizing with bevacizumab or radiotherapy [182, 183]. In the first-in-human phase I study (ISRCTN00759419), S49076 demonstrated limited single-agent activity with a tolerable safety profile at a recommended dose of 600 mg once daily in patients with advanced solid tumours [146]. S49076 was recommended for combination therapies; therefore, it is currently in phase I/II clinical trials in combination with gefitinib in MET/AXL-dysregulated NSCLC patients progressing on prior EGFR-TKI treatment [184].

##### Amuvatinib (MP-470; Astex Pharmaceuticals)

Amuvatinib, a multitargeted RTK inhibitor that targets AXL, KIT, and PDGFRα [140], was shown to disrupt DNA damage repair through the inhibition of Rad51 [185], to suppress AXL expression [186] and to resensitize cells to radio- and chemotherapies in GIST, lung cancer and glioblastoma cells [118, 140, 185–187]. MP-470 was recently reported to produce a limited response in a refractory GIST patient with KIT mutations and to show antitumour activity when combined with standard-of-care chemotherapy in neuroendocrine tumours, NSCLC, and small cell lung cancer (SCLC) [147, 188].

##### Sunitinib (SU11248, Sutent; Pfizer)

Sunitinib is an oral multi-targeted TKI with activity against PDGFR, VEGFR2, FLT3, KIT, and AXL [189–191]. It was the first drug jointly approved by the FDA for the treatment of both advanced RCC and imatinib-resistant/intolerant GIST in 2006 and was later approved for pancreatic neuroendocrine tumours (PNETs) [5, 192–195]. Resistance to sunitinib, whether intrinsic or acquired, still remains a challenge limiting its optimal clinical benefit [196]. Sunitinib is currently in clinical trials for multiple solid tumours, including thymic carcinoma, GIST, cholangiocarcinoma, urothelial carcinoma, NSCLC, soft tissue sarcoma and RCC (NCT01499121, NCT01498835, NCT00794950, NCT01718327, NCT01824615, NCT02623127, NCT03673501, and NCT00372775).

##### SNS314 (Sunesis Pharmaceuticals)

SNS314 is a pan-selective Aurora kinase inhibitor [197]. Although preclinical studies showed potent anti-tumor activity [198], results of a phase I clinical trial for the treatment of patients with advanced solid tumors were not satisfactory as no responses were observed. Thus, further development of SNS-314 was suspended [5].

##### Other type I AXL inhibitors in the preclinical stage

2-D08 is a unique inhibitor of protein sumoylation that has strong potency against AXL kinase with an in vitro IC50 of 0.49 nM [199]. 2-D08 is known to inhibit the phosphorylation of AKT and ERK, increase the expression of epithelial surfactant protein, and suppress EMT-mediating transcription factors, including SNAI2, HOXA5 and TBX2/3 [200]. In addition, the dual MER/FLT3 inhibitor UNC2025 [201], the selective AXL inhibitor SGI-7079 [202], and the novel MER and AXL inhibitor UNC569 [203, 204] have also been reported as promising molecules emerging from preclinical studies.

#### Type II AXL inhibitors (Table 2)

##### Cabozantinib (XL184, Cometriq®; Exelixis/Ipsen company)

Developed as a non-selective TKI that inhibits multiple RTKs, including MET, AXL, VEGFR2, RET, KIT, and ROS1 [205], and the related angiogenesis and metastasis processes [206, 207], cabozantinib has shown vast in vitro antitumour activity in extensive studies on human umbilical vein endothelial cells (HUVECs) and RCC, HCC, medullary thyroid cancer, and ovarian cancer cells [148, 208–214]. In vivo studies using xenograft models of breast cancer, lung cancer and glioma also showed the dose-dependent antitumour efficacy of cabozantinib [150, 206]. The capsule form of cabozantinib, Cometriq®, has been approved by the FDA for the treatment of advanced RCC and metastatic medullary thyroid carcinoma [215]. Numerous phase I/II trials have been conducted in patients with melanoma, metastatic breast cancer, NSCLC, AML, castration-resistant prostate cancer and HCC with promising preliminary activities [148–150, 216–220]. However, a randomized phase II study of 60 mg p.o. daily cabozantinib versus 80 mg/m^2^ paclitaxel weekly revealed that cabozantinib at this dose was not recommended over paclitaxel for the treatment of recurrent ovarian cancer [221]. Recently, the final results of the phase III CELESTIAL trial were published, which demonstrated a prolonged median OS for cabozantinib treatment compared with placebo (10.2 months vs. 8.0 months, hazard ratio (HR) = 0.76 (0.63–0.92), *p* = 0.005) [216]. Other phase III trials are now ongoing in differentiated thyroid cancer (NCT03690388), sorafenib pre-treated HCC (NCT01908426), and carcinoid tumours (NCT03375320).

**Table 2:** Summary of the basic profile of type II AXL inhibitors and the related ongoing clinical trials

Drug	Developer	Target(s)^a^	IC50 for AXL	Clinical Trial No.^b^	Phase of approval	Indications	Monotherapy/Combinations	Adverse events	Status
Cabozantinib (Cabometyx, XL184, BMS-907351, marketed as Cometriq)	Exelixis/Ipsen company	VEGFR2, MET, RET, KIT, AXL, FLT1/3/4	IC50 (in vitro) = 7 nM IC50 (in cells) = 42 nM	NCT01908426	III	Advanced HCC	Monotherapy	Fatigue, diarrhea, hypertension, palmar-plantar erythrodysesthesia syndrome	Completed
				NCT01865747	III	Advanced or metastatic RCC	Cabozantinib or Everolimus		Completed
				NCT01716715	II	Persistent or recurrent epithelial ovarian, fallopian tube, or primary peritoneal carcinoma	Cabozantinib vs. Paclitaxel		Completed
				NCT00596648	Ib/II	NSCLC	± Erlotinib		Completed
BMS-777607 (ASLAN002)	Bristol-Myers Squibb/Aslan Pharmaceuticals	AXL, RON, MET, Tyro3	IC50 (in vitro) = 1.1 nM	NCT00605618	I/II	Advanced solid tumors	Monotherapy	Not reported	Completed
				NCT01721148	I	Advanced solid tumors	Monotherapy		Completed
LY2801653 (Merestinib)	Eli Lilly and Company; Dana-Farber Cancer Institute	TEK, MET, AXL, RON	IC50 (in vitro) = 11 nM IC50 (in cells) = 2 nM	NCT02711553	II	Advanced or metastatic biliary tract cancer	Ramucirumab or merestinib or placebo, + cisplatin and gemcitabine	Not reported	Active, not recruiting
				NCT02920996	II	NSCLC	Monotherapy		Recruiting
				NCT03027284	I	Advanced and/or metastatic cancer	± Other anti-cancer agents		Active, not recruiting
Foretinib (XL880, EXEL-2880, GSK1363089)	GSK	MET, VEGFR2, TIE-2, VEGFR3, RON, AXL	IC50 (in vitro) = 11 nM IC50 (in cells) < 100 nM	NCT01147484	II	Recurrent breast cancer	Monotherapy	Fatigue, hypertension, gastrointestinal toxicities, nonfatal pulmonary emboli	Completed
				NCT00726323	II	RCC	Monotherapy		Completed
				NCT00920192	I	HCC	Monotherapy		Completed
MGCD516 (Sitravatinib)	Mirati Therapeutics Inc.	DDR2, EPHA3, AXL, MER, VEGFR3	IC50 (in vitro) = 1.5 nM IC50 (in cells) = 250–800 nM	NCT03680521	II	Clear cell RCC	+ Nivolumab	Not reported	Recruiting
				NCT02219711	I/Ib	Advanced solid tumors	Monotherapy		Recruiting
MGCD265 (Glesatinib)	Mirati Therapeutics	MET, RON, VEGFR1/2/3, AXL^c^	Not reported	NCT02544633	II	NSCLC with genetic alterations in MET	Monotherapy	Diarrhea, rash, fatigue	Completed
				NCT00697632	I	Advanced malignancies	Monotherapy		Completed
				NCT00975767	I	NSCLC	+ Erlotinib/docetaxel		Terminated
RXDX-106 (CEP-40783)	Ignyta, Inc.	AXL, MET, Tyro3, MER	IC50 (in vitro) = 7 nM	NCT03454243	I	Advanced or metastatic solid tumors	Monotherapy	Not reported	Terminated
Rebastinib (DCC-2036)	Deciphera Pharmaceuticals LLC	ABL, FLT3, VEGFR2, TIE-2, Lyn, SRC, FGR, AXL	IC50 (in vitro) = 42 nM	NCT03717415	I/II	Locally advanced or metastatic solid tumor	+ Carboplatin	Dry mouth, constipation, fatigue, muscular weakness, headache, nausea, blurred vision	Recruiting
				NCT00827138	I	CML	Monotherapy		Completed
NPS-1034	NeoPharm	AXL, MET	IC50 (in vitro) = 10.3 nM IC50 (in cells) < 0.5 μM	–	–	–	–	–	Preclinical
LDC1267	Lead Discovery Centre	MER, Tyro3, AXL	IC50 (in vitro) = 29 nM IC50 (in vivo) = ~ 15 μM	–	–	–	–	–	Preclinical

*Abbreviations*: *NSCLC* non-small cell lung cancer, *RCC* renal cell carcinoma, *CML* chronic myelogenous leukemia, *HCC* hepatocellular carcinoma

^a^In the order of inhibition potency

^b^All the relevant information of clinical trials can be found on the public clinical trial registry website (clinicaltrials.gov). Here is a partial list of all the relevant clinical trials

^c^Data for AXL not reported

##### BMS-777607 (ASLAN002; Bristol-Myers Squibb/Aslan Pharmaceuticals)

BMS-777607 was initially designed to inhibit MET kinase, but in fact, it was found to be a potent AXL kinase inhibitor with an IC50 of 1.1 nM in cell-free assays [222–224]. It significantly inhibits MET auto-phosphorylation; the activation of downstream molecules including ERK, AKT, p70S6K and S6; colony formation, migration; invasion; and HGF-induced cell scattering in GTL-16, H1993, U87, PC-3 and DU145 cells [142, 222, 225, 226]. This compound also demonstrates significant in vivo antitumour activity through increased apoptosis and decreased proliferation and migration in the GTL-16 human gastric carcinoma xenograft model [222]. In a KHT sarcoma rodent tumour model, BMS-777607 impaired metastasis [225], led to the regression of intracranial glioma tumour growth and reduced AXL-related tumour angiogenesis [43]. In addition, BMS-777607 is a potent polyploidy inducer that promotes the megakaryocytic differentiation of CHRF-288-11 cells [227]. This compound is now undergoing phase I/II clinical trials in patients with advanced or metastatic tumours (NCT01721148 and NCT00605618).

##### LY2801653 (Merestinib; Eli Lilly and Company/Dana-Farber Cancer Institute)

LY2801653 is a dual MET/AXL inhibitor that targets RON, MET, and AXL [228, 229]. LY2801653 potently blocks the phosphorylation of MET and AXL and the activation of their downstream signalling molecules. In cholangiocarcinoma, LY2801653 inhibits migration, invasion, colony formation, and concomitant in vivo tumour growth through the suppression of MET and downstream targets [230]. LY2801653 disrupts the activity of mesenchymal glioma stem cells through the inhibition of MAPK-interacting kinases (MNKs) [73] and has a demonstrated antitumour effect in xenograft models of AML, gastric cancer, cholangiocarcinoma and lung cancer cells [229, 231, 232]. LY2801653 is now being investigated in patients with breast cancer, AML, biliary tract cancer and NSCLC (NCT03027284, NCT01285037, NCT03125239, NCT03292536, and NCT02711553).

##### Foretinib (XL880, EXEL-2880, GSK1363089; GSK)

Foretinib is an oral multi-kinase inhibitor of AXL, MET, VEGFR, ROS, RON, and TIE-2 [161, 233]. Foretinib blocks AXL phosphorylation and is associated with suppressed cell proliferation, dissemination and survival and the inhibition of in vivo tumour growth and peritoneal metastasis in an orthotopic colorectal cancer xenograft model [234]. In HER2-positive breast cancer cells that overexpress AXL, treatment with foretinib in combination with HER2-targeted therapies renders cells more vulnerable to lapatinib [123]. In a phase I clinical trial, the observation of three confirmed partial responses and 22 cases of stable disease in a total of 40 patients confirmed the antitumour activity of foretinib [235]. Data from phase I/II studies showed evidence of tumour regression in patients with advanced papillary renal cell carcinoma (PRCC), TNBC, NSCLC and HCC [151, 153, 236, 237]. However, in a recent phase II study evaluating foretinib in gastric cancer, single-agent foretinib lacked efficacy even in MET-amplified patients with metastatic gastric cancer [152], which demonstrated the requirements for ascertaining the mechanisms of gastric cancer oncogenesis and molecular patient selection. The toxicity profile was relatively manageable, and hypertension and elevated aspartate aminotransferase (AST) were common side effects in patients with cancer [151–153, 235–237].

##### MGCD516 (Sitravatinib; Mirati Therapeutics Inc.)

MGCD516 blocks a closely related spectrum of RTKs, including KIT, PDGFRβ, PDGFRα, MET, and AXL [238]. MGCD516 demonstrates better in vitro and in vivo efficacy in sarcoma cell lines than two well-known TKIs, imatinib and crizotinib, and augments immune checkpoint blockade in unresponsive tumours [238, 239]. A phase I trial with MGCD516 is now recruiting patients with solid tumours (NCT02219711).

##### MGCD265 (Glesatinib; Mirati Therapeutics)

MGCD265 is a small molecule multi-targeted TKI that targets MET, VEGFR1/2/3, RON, TIE-2 and AXL [240], and it has been shown to have a potent clinical response in patients with metastatic NSCLC with AXL amplification [241]. Phase I/II clinical trials in patients with metastatic NSCLC harbouring genetic alterations in MET and advanced malignancies [242] have been completed, but the results are not yet available.

##### RXDX-106 (Ignyta, Inc.)

RXDX-106 is a selective and potent pan-TAM family inhibitor that exerts antitumour efficacy through regulating immune cells, including M1-polarized intra-tumoural macrophages, NK cells, CD8+ T cells and dendritic cells, and may lead to suppressed tumour growth and progression [243].

##### Rebastinib (DCC-2036; Deciphera Pharmaceuticals LLC)

Rebastinib, designed as a switch-control inhibitor of the BCR-ABL1 tyrosine kinase [244], also has striking activity against AXL in TNBC cells [245]. It is now in phase I/II clinical trials for the treatment of locally advanced or metastatic solid tumours, CML and breast cancer (NCT03717415, NCT03601897, NCT00827138, and NCT02824575).

##### Other type II inhibitors at the preclinical stage

NPS-1034 is a newly developed dual AXL/MET inhibitor that exerts efficacy against cancer cells harbouring activated or mutated *MET* or *AXL* [246, 247]. LDC1267, a highly selective TAM kinase inhibitor, is able to awaken the innate immune system and enhance NK cell activity to kill cancer metastases in vivo [127].

### Anti-AXL mAbs (Table 3)

Current AXL molecular targeted therapeutics exhibit either modest antitumour efficacy, cellular cytotoxicity, or significant off-target effects [46, 229, 248], which prompted the emergence of high affinity anti-AXL mAbs. YW327.6S2 is a blocking antibody that binds to both human and murine AXL, limiting receptor activation and downstream signalling through the ligand Gas6. YW327.6S2 attenuates tumour growth, metastasis, angiogenesis and the secretion of inflammatory cytokines and chemokines from tumour-associated macrophages (TAMs) and potentiates the efficacy of chemotherapy and other small-molecule inhibitors [139].

**Table 3:** Anti-AXL monoclonal antibodies and nucleotide aptamers currently being investigated

Name	Type	Target	Indications	Phase of clinical trials
YW327.6S2	Monoclonal antibody	AXL	NSCLC, breast cancer	Preclinical
D9	Monoclonal antibody	AXL	Pancreatic cancer	Preclinical
E8	Monoclonal antibody	AXL	Pancreatic cancer	Preclinical
MAb173	Monoclonal antibody	AXL	Kaposi sarcoma	Preclinical
AXL-107-MMAE	Antibody-drug conjugate	AXL	Melanoma	Preclinical
^64^Cu-anti-hAXL	64Cu-labeled anti-human antibody	AXL	Breast cancer	Preclinical
Axl specific CAR and SynNotch receptor	CAR and synNotch receptors	AXL	Leukemia	Preclinical
GL21.T	RNA nucleotide aptamer	AXL	NSCLC	Preclinical
GL21.T/miR-34c chimera	conjugate of miR-34c and GL21.T	AXL	NSCLC	Preclinical
DNA AXL-APTAMER	DNA nucleotide aptamer	AXL	Ovarian cancer	Preclinical

*Abbreviation*: *NSCLC* non-small cell lung cancer

Two other selected anti-AXL mAbs, D9 and E8, are efficient in inhibiting the proliferation and migration of pancreatic cancer cells through blocking the phosphorylation of AXL and downstream molecules without affecting GAS6 binding [249]. Other anti-AXL mAbs include 20G7-D9 [84], MAb173 [250], ^64^Cu-labelled anti-AXL antibody [251], antibody-based agents such as the antibody-drug conjugate (ADC) AXL-107-MMAE [252] and AXL-specific CAR and SynNotch receptor [253], which have also shown promising results in preclinical studies.

### Nucleotide aptamers (Table 3)

Nucleotide aptamers are immerging alternatives with higher affinity and lower toxicity than current standard therapies [248, 249, 254]. Aptamers are short structured single-stranded RNAs or DNAs that can act as ligands by binding to their targets. Their low cost, convenient generation, low immunogenicity, sufficient stability, and potential as targeted delivery tools for nanoparticles, chemotherapeutics or siRNAs make them promising therapeutics in neoplastic diseases [255, 256].

The RNA aptamer GL21.T was designed to specifically recognize the extracellular domain of AXL. It hampers AXL-dependent downstream ERK and AKT phosphorylation; interferes with cell migration, invasion and colony formation; and inhibits in vivo tumour growth in a mouse xenograft model of human NSCLC cells [255]. The conjugate of miR-34c and the GL21.T aptamer, GL21.T/miR-34c, exhibits dual functional and transcriptional inhibition of AXL in NSCLC cells [257]. Based on the sequence of GL21.T, the corresponding DNA aptamer was synthesized and was more resistant to hydrolysis. This DNA AXL-APTAMER could inhibit AXL phosphorylation and the related cell proliferation in vitro and in vivo and potentiate chemotherapy efficacy in ovarian cancer models [42].

### Soluble receptors

Since the TAM family members conventionally undergo alternative splicing or proteolytic cleavage of extracellular domains [10], these soluble extracellular domains produced might act as a ligand sink to downregulate the receptor [258]. The soluble ectodomain of AXL, termed soluble AXL (International Patent application WO2008098139), acts as a ‘decoy receptor’ that binds GAS6, abrogating AXL signalling and Gas6-induced mitogenic effects, and has shown promising results in animal models of metastasis [10, 255, 259, 260]. In addition, soluble AXL has considerable potential as a diagnostic marker in patients at early stage HCC and cirrhosis [261].

### Other natural compounds as inhibitors of AXL

Natural compounds could also function as inhibitors of AXL. For example, celastrol exhibits a synergistic effect with gefitinib in suppressing cell proliferation and migration and increases the susceptibility of EGFR-mutant NSCLC cells to gefitinib [262]. Dihydroartemisinin (DHA), the active derivative of the well-known anti-malarial drug artemisinin, blocks AXL expression and the related proliferation, migration, and tumour development of prostate cancer cells via the miR-34a/miR-7/JARID2 pathway [263]. Recently, a new class of quinolone-based compounds has emerged as selective AXL inhibitors that could inhibit TGF-â1-induced MDA-MD-231 breast cancer cell migration and invasion in a dose-dependent manner [264]. In addition, mistletoe extract of *Viscum album* extract (VAE) was reported to inhibit AXL expression, suppress cell proliferation and overcome cisplatin- and erlotinib-resistance in NSCLC cells [265].

## Future perspectives and conclusion

Although AXL-targeted therapies appear promising in treating malignancies, many questions remain unanswered. AXL small molecule inhibitors have pronounced therapeutic effects; nonetheless, their inherent limitations, the off-target effects, might give rise to the inhibition of additional kinases and subsequent unexpected toxicities, limiting their clinical use. A variety of side effects have been reported for molecular targeted therapies, especially for multi-targeted AXL TKIs [148, 151–153, 210, 220, 235–237].

In addition, many patients do not respond to anti-AXL treatment or acquire resistance to these agents. Therefore, it is beneficial to select the optimal patients who could draw clinical benefits from AXL inhibitors and to avoid potential toxicities. Reliable integrated biomarker design is warranted to guide treatment strategies in predicting response and overcoming therapeutic resistance.

AXL inhibitors, either as single agents or in combination with conventional chemotherapy or other inhibitors such as immune checkpoint inhibitors, angiogenesis inhibitors and other TKIs, are likely to improve the survival of many patients. However, rational combination approaches, the sequence of administration, and the right time of incorporation of anti-AXL agents into treatment regimens should be taken into consideration before clinical use. Dissection of AXL molecular signalling networks and further investigations into the relationships between AXL and other kinases need to be performed to improve antitumour therapies and personalized cancer treatment.

## Data Availability

The datasets used and/or analysed during the current study are available from the corresponding author on reasonable request.

## Abbreviations

TKIs: Tyrosine kinase inhibitors
RTKs: Receptor tyrosine kinases
*Gas6*: Growth arrest specific gene 6
GAS6: Growth arrest-specific protein 6
EGFR: Epidermal growth factor receptor
VEGF: Vascular endothelial growth factor
PDGF: Platelet-derived growth factor
PDGFR: Platelet-derived growth factor receptor
FGF: Fibroblast growth factor
FGFR: Fibroblast growth factor receptor
PI3K: PHOSPHATIDYLINOSITOL 3-kinase
PAK1: PI3K/AKT-P21-activated kinases-1
HER2: Human epidermal growth factor receptor 2
EMT: Epithelial mesenchymal transition
mAbs: Monoclonal antibodies
FLT3: FMS-like Tyrosine Kinase 3
MNKs: MAPK-interacting kinases
Fro III: Fibronectin type III
Ig: Immunoglobulin
NCAMs: Neural cell adhesion molecules
AP1: Activator protein 1
HIF1α: Hypoxia inducible factor 1α
MZF-1: Myeloid zinc finger 1 protein
TULP-1: Tubby-like protein 1
SH2: Src homology 2
PTBs: Phosphotyrosine-binding domains
miRNAs: microRNAs
shRNA: short hairpin RNA
siRNA: Small interfering RNA
RNAi: RNA interference
HUVECs: Human umbilical vein endothelial cells
TAMs: Tumor-associated macrophages
ADC: Antibody-drug conjugate
DHA: Dihydroartemisinin
VAE: Visucm album extract
SCLC: Small cell lung cancer
NSCLC: Non-small cell lung cancer
HNSCC: Head and neck squamous cell carcinoma
AML: Acute myeloid leukemia
CLL: Chronic lymphocytic leukemia
CML: Chronic myeloid leukemia
HCC: Hepatocellular carcinoma
RCC: Renal cell carcinoma
PRCC: Papillary renal cell carcinoma
GIST: Gastrointestinal stromal tumor
MDS: Myelodysplastic syndromes
TNBC: Triple-negative breast cancer
ALCL: Anaplastic large cell lymphoma
PNETs: Pancreatic neuroendocrine tumors
OS: Overall survival
HR: Hazard ratio
AST: Aspartate aminotransferase
PDXs: Patient-derived xenografts
